# Prediction of bile duct injury after transarterial chemoembolization for hepatocellular carcinoma: Model establishment and verification

**DOI:** 10.3389/fonc.2022.973045

**Published:** 2022-12-16

**Authors:** Jianxi Guo, Xueying Zhang, Jian Kong

**Affiliations:** Department of Interventional Radiology, Shenzhen People’s Hospital (Second Clinical Medical College of Jinan University, First Affiliated Hospital of Southern University of Science and Technology), Shenzhen, China

**Keywords:** DEB-TACE, HCC, bile duct injury, risk factors, prediction model

## Abstract

**Objective:**

This study aimed to establish and validate a predictive model for bile duct injury in patients with hepatocellular carcinoma (HCC) after drug-eluting bead transarterial chemoembolization (DEB-TACE).

**Methods:**

We retrospectively analyzed 284 patients with HCC treated with DEB-TACE at our hospital between January 2017 and December 2021, of whom 63 patients experienced postoperative bile duct injuries. Univariate and logistic multivariate regression analyses were performed to identify the risk factors for bile duct injury, as well as establish and internally validate the nomogram model. The area under the curve (AUC) of the receiver operating characteristic (ROC) curve, calibration curve, Hosmer-Lemeshow goodness of fit test, decision curve analysis (DCA), and clinical impact curve (CIC) were used to assess the predictive power, clinical value, and practicability of the nomogram model.

**Results:**

The incidence of bile duct injuries after DEB-TACE was 22.18% (63/284), with one injury occurring in every 2.86 sessions of DEB-TACE treatment. Univariate and logistic multivariate regression analyses indicated that a history of hepatectomy (odds ratio [OR]=2.285; 95% confidence interval [CI]=1.066–4.898; P<0.05), subjective angiographic chemoembolization endpoint level (OR=1.832; 95% CI=1.258–2.667; P<0.05), alkaline phosphatase (OR=1.005; 95% CI=1.001–1.010; P<0.05), and platelet count (OR=1.005; 95% CI=1.001–1.009; P<0.05) were independent risk factors for bile duct injury after DEB-TACE among patients with HCC. The risk nomogram model based on the above four variables was validated using the bootstrap method, showing consistency between the predicted and experimental values. Furthermore, the model performed well in the Hosmer-Lemeshow goodness-of-fit test (^2^=3.648; P=0.887). The AUC of this model was 0.749 (95% CI=0.682–0.817), with an overall accuracy of 69.01%, a positive predictive value of 73.02%, a negative predictive value of 67.87%, a sensitivity of 73.0%, and a specificity of 67.90%, suggesting that the nomogram model had good accuracy and discrimination. In addition, DCA and CIC revealed a high clinical value and practicability of the model.

**Conclusion:**

Bile duct injury in patients with HCC treated with DEB-TACE is caused by multiple factors rather than a single factor. The nomogram prediction model used in this study had a good fitting degree and prediction efficacy, with high clinical value and practicability.

## Introduction

Transarterial chemoembolization (TACE) is the recommended treatment for intermediate-stage hepatocellular carcinoma (HCC), postoperative recurrence of HCC, and a few cases of stage A Barcelona Clinic Liver Cancer (BCLC) because of its advantages such as minimal invasiveness, safety, and fewer complications (1). Previous studies have shown that some patients with HCC can achieve the standard of clinical cure or long-term tumor-bearing survival, as well as a significant improvement in the quality of life after treatment with TACE (2, 3). Ischemic bile duct injury, also known as ischemic biliary tract disease, ischemia-related biliary tract disease, or ischemic cholangitis, is a complex biliary tract disease that refers to a local or diffuse bile duct injury in which various factors cause bile duct damage (4). Bile duct injury is a rare and serious complication of conventional TACE (c-TACE) with an estimated incidence of 0.5–4% (5–8). In recent years, the increasing demand for TACE led to the development of novel embolization agents with improved therapeutic effects and fewer systemic adverse effects; thus, drug-eluting beads TACE (DEB-TACE) is gradually being applied in clinical practice. Several retrospective and prospective clinical studies have documented a higher incidence of postoperative ischemic bile duct injury and other complications with DEB-TACE than with c-TACE (9–11). Due to the lack of effective approaches, treatment outcomes and prognosis of ischemic bile duct injury remained unsatisfactory (12). Several studies have described the pathophysiology, risk factors, imaging findings, and clinical significance of bile duct injuries (13, 14); however, predictive factors of bile duct injury after DEB-TACE have received little attention. Therefore, this retrospective study analyzed the incidence of bile duct injury after DEB-TACE and by screening potential risk factors, we developed a nomogram prediction model for postoperative bile duct injury among patients with HCC undergoing DEB-TACE.

## Material and methods

### Study population

The institutional review board of Shenzhen People’s Hospital approved this retrospective study (No. LL-KY-2022137-01) and waived the requirement for informed consent from the patients. All the procedures performed in this study involving human participants were in accordance with the 2013 revision of the Declaration of Helsinki.

A total of 284 patients diagnosed with HCC were treated with DEB-TACE at our hospital between January 2017 and December 2021, and the patients are consecutive in the present study. The inclusion criteria were as follows: 1) pathological or clinically confirmed diagnosis of HCC according to the American Association for the Study of Liver Diseases; 2) liver function grade A or B in Child–Pugh class, stage A or B BCLC, and 0 score in Eastern Cooperative Oncology Group; patients who refused to undergo surgery and those with no surgical indications or postoperative residual disease and recurrence but consistent with DEB-TACE treatment indicators; 3) those who did not undergo other treatments for HCC before hepatectomy or DEB-TACE treatment until the follow-up period, including interventional therapy (e.g., c-TACE and ablation), chemotherapy, and radiotherapy; 4) no bile duct injury before hepatectomy or DEB-TACE; and 5) DEB-TACE treatment performed for ≥2 times with a follow-up period of ≥6 months or those who achieved a complete response (CR) after initial DEB-TACE treatment after a follow-up period of ≥6 months. Patients were excluded if 1) those with a history of choledochojejunostomy, percutaneous transhepatic cholangiodrainage, endoscopic retrograde cholangiopancreatography, or other biliary system surgeries; 2) patients with infiltrative HCC; 3) those with missing data or who did not complete the follow-up; 4) patients with comorbid severe heart, lung, liver, kidney, or other organ dysfunction; and 5) those with contraindications for vascular interventional procedures, such as severe coagulopathy and iodine allergy.

### DEB-TACE procedure

All interventional procedures were performed in an independent room of digital subtraction angiography (DSA,1200 mA, Siemens, Munich, Germany), and all interventional radiologists completed specialized training in interventional radiology with more than 10 years of experience in independent procedures. DEB-TACE was performed as follows: the patient was placed in the supine position, and the inguinal region was disinfected and draped. The puncture site was anesthetized using local anesthesia; the right femoral artery was punctured using the Seldinger method, and a 5F catheter sheath (Terumo, Tokyo, Japan) was passed through the artery. A 5F-Pigtail (Cordis, Miami Lakes, USA) catheter was introduced using a guidewire (Terumo, Tokyo, Japan) for abdominal aortography to observe the presence of variant and parasitic vessels supplying the tumor area. A Yashiro catheter (Terumo, Tokyo, Japan) was passed through the celiac trunk or the common hepatic artery for angiography. Angiography image collection included the arterial, parenchymal, and venous phases. Attention was paid to identifying the collateral feeding artery of the tumor to assess the blood flow in the tumor-supplying artery and portal vein and the presence of a combined hepatic arteriovenous fistula. A 2.4F coaxial microcatheter (Boston Scientific, Boston, MA, USA) was superselectively passed through the segmental or subsegmental tumor-supplying artery while avoiding the cystic, right gastric, and sickle arteries. The diameter of the CalliSheres ^®^ beads (Hengrui Medical, Suzhou, China) was selected according to the tumor size and number and intraoperative angiographic findings. CalliSheres^®^ beads were loaded with chemotherapeutic drugs (2 mL/vial DEBs with 50 mg pirarubicin), which were then mixed with 10–15 mL non-ionic contrast agent, iophorol-350 (Hengrui Medical, Suzhou, China). Sterile water and/or a contrast agent was injected to achieve a good suspension of the microspheres. After the catheter reached the target vessel, the micropheres injected slowly at a rate of 1 mL/min using a 3 mL syringe. Subjective angiographic chemoembolization endpoint (SACE) level was used to assess embolization, and the ideal endpoint was defined as the disappearance of residual tumor blush and near stasis of the residual antegrade arterial flow. Repeated angiography was performed 5 min later to confirm the embolization endpoint. The catheter and sheath were removed, compression hemostasis and bandaging were performed at the puncture site, and the right lower limb was immobilized in bed for 8 h. All patients were routinely administered symptomatic treatment for liver and stomach protection and pain relief, as well as antiemetic therapy after procedure.

### Data collection

The data of all patients were collected from the inpatient information retrieval system, including 1) demographic data: age, sex, personal history, medical history, comorbidity, and treatment history; 2) clinical features: tumor load, size, and distribution; 3) imaging data: abdominal ultrasonography (US), computed tomography (CT), and magnetic resonance imaging (MRI) before and after bile duct injury; 4) laboratory examination: routine blood tests, coagulation function, liver and kidney function, and tumor markers; 5) the number of DEB-TACE treatments; and 6) SACE level (15). The four stages of SACE are SACE I: normal residual antegrade arterial flow and reduced residual tumor blush; SACE II: reduced residual antegrade arterial flow and residual tumor blush; SACE III: reduced residual antegrade arterial flow and no residual tumor blush; and SACE IV: no residual antegrade arterial flow and residual tumor blush. If DEB-TACE was performed more than once, follow-up was continued until bile duct injury was detected.

### Imaging assessment of bile duct injury

Imaging evaluation was completed by two associated chief radiologists who have been engaged in CT and MRI diagnosis for more than 10 years, and both were blinded to the patient’s baseline data and treatments. Disagreements were resolved through consultation. If the bile duct was normal before DEB-TACE, the imaging manifestations of the bile duct injury after the procedure, such as intrahepatic biliary dilatation, biloma, and hilar biliary stricture, were recorded (5, 16, 17). Specific imaging findings were as follows: 1) imaging diagnosis of bile duct dilatation revealed bile duct shadows distributed in the liver lobe or a segment along the Glisson’s sheath and running along the portal vein, which might be accompanied by congestion and edema; 2) imaging diagnosis of the hilar biliary stricture was based on magnetic resonance cholangiopancreatography, and the original MRI images showed hilar bile duct filling defect performance or different degrees of stenosis with or without bile duct dilatation; 3) imaging diagnosis of biloma revealed circular, isolated, or polycystic low-density areas accompanied by signs of infection or quasi-circular low-density areas distributed along the Glisson’s sheath, which communicated with the bile duct. Bile duct injury caused by DEB-TACE was defined as any one or more of the aforementioned imaging findings combined with laboratory tests of AKP, GGTP, TBil and DBil (14). It should also be differentiated from bile duct changes due to tumor invasion, include imaging manifestations, history of TACE treatment, morphology and occurrence time of bile duct dilatation.

### Follow-up protocol

All patients underwent routine blood tests, coagulation function, liver and kidney function, and imaging examinations (e.g., CT, MRI, and US) before DEB-TACE. The first follow-up was conducted by performing CT/MRI and enhanced CT/MRI 4–6 weeks after the procedure. If the tumor response was evaluated as complete response (CR), the follow-up was performed every 2–3 months and then every 6 months; however, if the tumor response was evaluated as a partial response (PR), DEB-TACE treatment was continued according to the treatment plan until CR was achieved. If tumor recurrence or progression occurred during follow-up, the corresponding treatment regimen was administered according to the BCLC criteria. All patients were followed up for at least 6 months after the initial DEB-TACE treatment, and the follow-up was terminated when patients experienced bile duct injury or serious complications.

### Statistical analysis

The kappa index was calculated to analyze the consistency between the two radiologists, with values <0.4 indicating poor agreement, 0.4–0.75 indicating good agreement, and >0.75 indicating excellent agreement. Continuous variables are expressed as mean ± standard deviation for normally distributed and non-normally distributed variables. Numerical differences between the two groups were assessed using the chi-square test for categorical variables, and continuous variables were assessed using the t-test or Mann–Whitney U test. Univariate analysis was used to screen for potential risk factors for bile duct injury, and multivariate logistic regression analysis was performed. A p-value of < 0.05 was considered statistically significant. Statistical analyses were performed using SPSS, version 26.0 (IBM Corp., Armonk, NY, USA).

The RMS program was used to establish the risk nomogram prediction model, and the Caret program bootstrap method was used for internal verification. ROCR and RMS program were used to draw the receiver operating characteristic (ROC) curves. The area under the curve (AUC), accuracy, positive and negative predictive values, sensitivity, and specificity of the ROC curve were calculated to assess the accuracy and discrimination of the model. The calibration curve and Hosmer-Lemeshow goodness-of-fit test were developed to evaluate the consistency of the observed and theoretical values, and P >0.05 indicated good performance. Clinical impact curve (CIC) and decision curve analysis (DCA) were performed to reflect the net benefit and clinical practicability of the nomogram model. The above statistical analyses were conducted using R software, version 3.6.1.

## Results

### Basic characteristics

**Figure 1:**
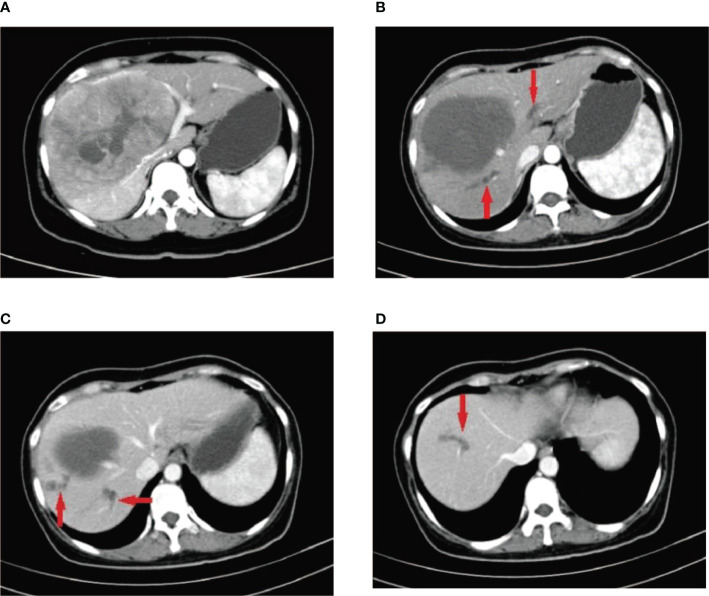
A 41-year-old female patient who was diagnosed with hepatitis B more than 10 years ago underwent CT examination due to epigastric pain, and CT findings indicated a large hepatocellular carcinoma in the right lobe of the liver **(A)**. The patient underwent two DEB-TACE treatment sessions. After 6 weeks, CT reexamination revealed a significant reduction in necrosis of the liver tumor; however, multiple intrahepatic bile duct necroses were observed around the tumor without significant enhancement **(B–D)** (red arrow).

**Figure 2:**
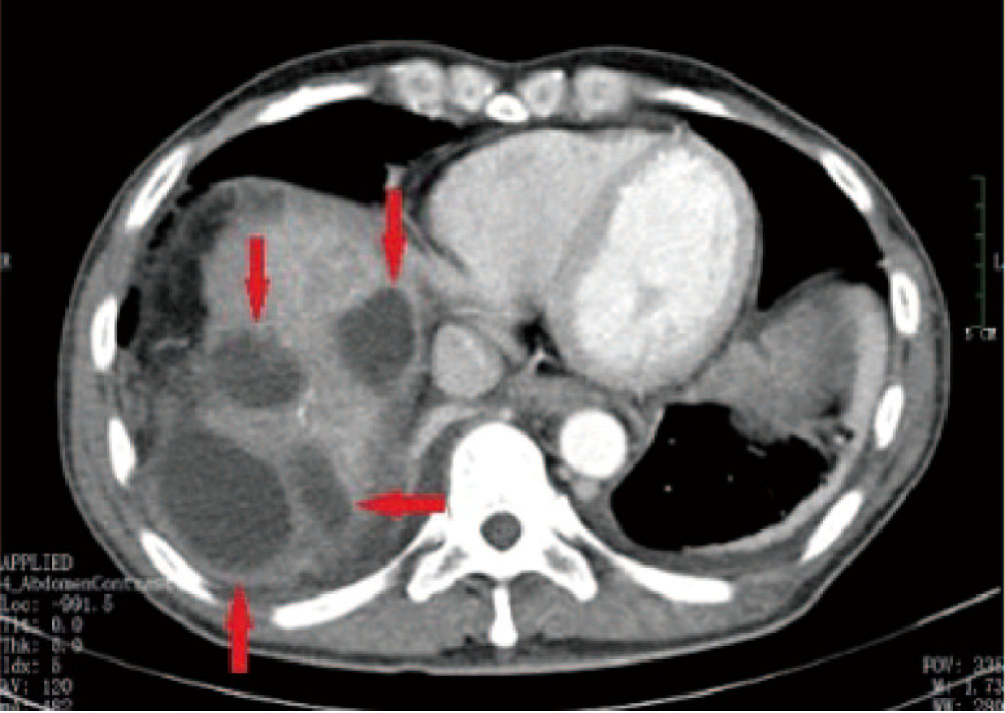
A 52-year-old male patient with HCC who received multiple DEB-TACE treatment sessions complained of abdominal pain accompanied by high a fever 4 weeks after undergoing the procedure. CT findings indicated multiple low-density shadows in the liver without obvious enhancement (red arrow). During puncture catheter drainage, we found that the drainage was a clear, yellowish biliary fluid, not liver abscess suppurative fluid, and the bacterial culture of the fluid was negative on multiple occasions.

**Figure 3:**
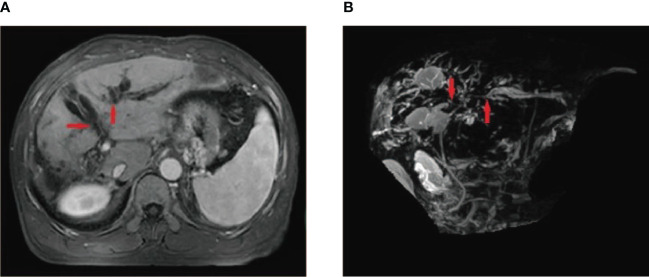
A 55-year-old male patient who underwent HCC resection and received two DEB-TACE treatment sessions presented with mild yellow staining of the sclera and skin, which occurred 6 weeks after the procedure. MRI and MRCP findings suggested intrahepatic bile duct dilation, localized stricture of the hilar bile duct, and no signs of tumor invasion or compression in the hilar liver **(A, B)** (red arrow).

A total of 284 patients with HCC were included, and an average of 2.82 ± 1.35 (range, 1–7) DEB-TACE treatment sessions per patient were performed successfully, with a total number of 802 DEB-TACE sessions. Varying degrees of postoperative bile duct injury occurred in 63 patients (22.18%), including 41 (65.08%) with biliary dilatation (**Figure 1**), 12 (19.05%) with biloma (**Figure 2**), and 10 (15.87%) with hilar biliary strictures (**Figure 3**). The mean age was 54.67 ± 11.94 years, and there were 43 (68.25%) males and 20 (31.75%) females. Of the 63 patients with a bile duct injury, 48 (76.19%) and 7 (11.11%) patients had HBV and HCV infections, respectively, 8 (12.70%) had alcohol/other types of cirrhosis, and 16 (25.40%) had a history of liver tumorectomy. There were 48 (76.19%) patients with grade A and 15 (23.81%) patients with grade B liver function Child–Pugh scores. As for BCLC, 33 (52.38%) patients had stage A and 30 (47.62%) had stage B BCLC (**Table 1**).

**Table 1:** Demographic characteristics and results of the univariate analysis of patients with HCC.

Variables	Bile duct injury (n=63)	No bile duct injury (n=221)	T/χ^2^/Z value	P value
Gender				
Males/Females	43/20	145/76	0.153	0.409
Age	54.67 ± 11.94	53.13 ± 14.34	0.778	0.025
Liver disease background				
HBV/HCV/Alcohol/Others	48/7/6/2	173/24/15/9	0.629	0.787
History of hepatectomy				
Yes/No	16/47	30/191	5.048	0.023
TACE number	2.86 ± 1.28	2.81 ± 1.27	0.221	0.591
TACE interval time(d)	40.57 ± 8.68	40.64 ± 8.051	0.061	0.333
Tumor number	2.75 ± 1.98	3.19 ± 2.32	1.396	0.069
Tumor diameter(mm)	56.16 ± 31.51	61.37 ± 33.82	1.905	0.285
Tumor burden(6-12 score)	8.41 ± 3.51	9.43 ± 3.91	1.870	0.191
Tumor burden group				
≤6/6-12/≥12	19/34/10	62/98/61	4.862	0.028
BCLC stage				
A/B	33/30	103/108	0.655	0.252
CP class				
A/B	48/15	147/74	2.133	0.094
SACE level				
I/II/III/IV	5/9/30/19	27/90/76/28	21.987	0.000
Bead diameter(µm)				
70-150/100-300/300-500	20/33/10	42/130/49	4.937	0.041
ALT	51.19 ± 45.75	53.45 ± 47.09	0.338	0.736
ASL	69.94 ± 57.77	59.32 ± 51.12	1.412	0.159
AKP	182.24 ± 140.75	129.26 ± 70.73	4.082	0.000
GGTP	215.87 ± 280.62	147.18 ± 128.96	2.764	0.006
ALB	35.64 ± 4.99	34.871 ± 5.27	1.032	0.303
TBil	30.07 ± 39.34	27.23 ± 31.77	0.590	0.555
DBil	17.82 ± 30.82	15.66 ± 25.48	0.567	0.571
PLT	164.38 ± 92.85	129.76 ± 82.15	2.865	0.004
PT	13.05 ± 1.59	13.305 ± 1.55	1.124	0.262
PT extension time	1.17 ± 1.55	1.442 ± 1.65	1.163	0.246

CP, Child-Pugh score; HBV, hepatitis B virus; HCV, hepatitis C virus.

### Treatment response

The kappa consistency test was used to analyze the inter-examiner agreement in the diagnosis of bile duct injury after DEB-TACE, and the kappa value was 0.85, indicating excellent consistency.

The mean maximum tumor diameter was 56.16 ± 31.51 mm, and the mean number of tumors was 2.75 ± 1.98 among the 63 patients with HCC. Patients were grouped according to tumor burden ranging from six-and-twelve score (linear predictor = largest tumour diameter (cm) + tumour number) (18), including 19 (30.16%) patients with a tumor burden of ≤6, 34 (53.97%) with a tumor burden of 6–12, and 10 (15.87%) with a tumor burden of ≥12. These 63 patients with bile duct injury underwent a total of 180 DEB-TACE sessions, with an average interval of 40.57 ± 8.68 days between two DEB-TACE treatments. One bile duct injury occurred in every 2.86 DEB-TACE treatment sessions and 56.13 ± 47.72 days after the last treatment. Overall, 5 (7.94%) patients had SACE I, 9 (14.28%) had SACE II, 30 (47.62%) had SACE III, and 19 (30.16%) had SACE IV. The diameter of the loaded beads selected for DEB-TACE treatments ranged from 70–150 µm in 20 (31.75%) patients, 100–300 µm in 33 (52.38%), and 300–500 µm in 10 (15.87%) patients (**Table 1**).

### Risk factors of bile duct injury

Univariate analysis showed that there were significant differences in age, history of hepatectomy, tumor burden group, SACE level, Bead diameter, alkaline phosphatase (AKP), gamma glutamyl-transpeptidase (GGTP), and platelet (PLT) count between HCC patients with and without bile duct injury after DEB-TACE (all P <0.05; **Table 1**).

The abovementioned factors with statistical significance were included in the multivariate analysis. As shown in **Table 2**, a history of hepatectomy (odds ratio [OR], 2.285; 95% confidence interval [CI], 1.066–4.898; P <0.05), SACE level (OR, 1.832; 95% CI, 1.258–2.667; P <0.05), AKP (OR, 1.005; 95% CI, 1.001–1.010; P <0.05), and PLT count (OR, 1.005; 95% CI, 1.001–1.009; P < 0.05) were all independent risk factors for bile duct injury after DEB-TACE. Specifically, in the stratified analysis of SACE levels, no significant difference (P >0.05) was found in SACE I between patients with bile duct injury (5 injuries, 7.94%) and without bile duct injury (27 cases, 12.22%); similar results were found in SACE III with p values >0.05. In contrast, among patients with SACE II, the proportion of bile duct injuries (9 injuries, 14.28% vs 90 cases, 40.72%) was significantly lower (P <0.05). Among patients with SACE IV, the proportion of bile duct injuries (19 injuries, 30.16% vs 28 cases, 12.67%) was significantly higher (P <0.05).

**Table 2:** Results of the multivariate logistic regression analysis.

Variables	B	SE	Waldχ^2^	P	OR	95%CI
Age	0.014	0.012	1.471	0.225	1.014	0.991-1.038
History of hepatectomy	0.826	0.389	4.511	0.034	2.285	1.066-4.898
Tumor burden group	-0.320	0.334	0.917	0.338	0.726	0.377-1.398
SACE level	0.605	0.192	9.979	0.002	1.832	1.258-2.667
Bead diameter	-0.283	0.388	0.531	0.466	0.754	0.352-1.613
AKP	0.005	0.002	4.981	0.026	1.005	1.001-1.010
GGTP	0.000	0.001	0.011	0.918	1.000	0.998-1.003
PLT	0.005	0.002	7.133	0.008	1.005	1.001-1.009

### Risk nomogram model

**Figure 4:**
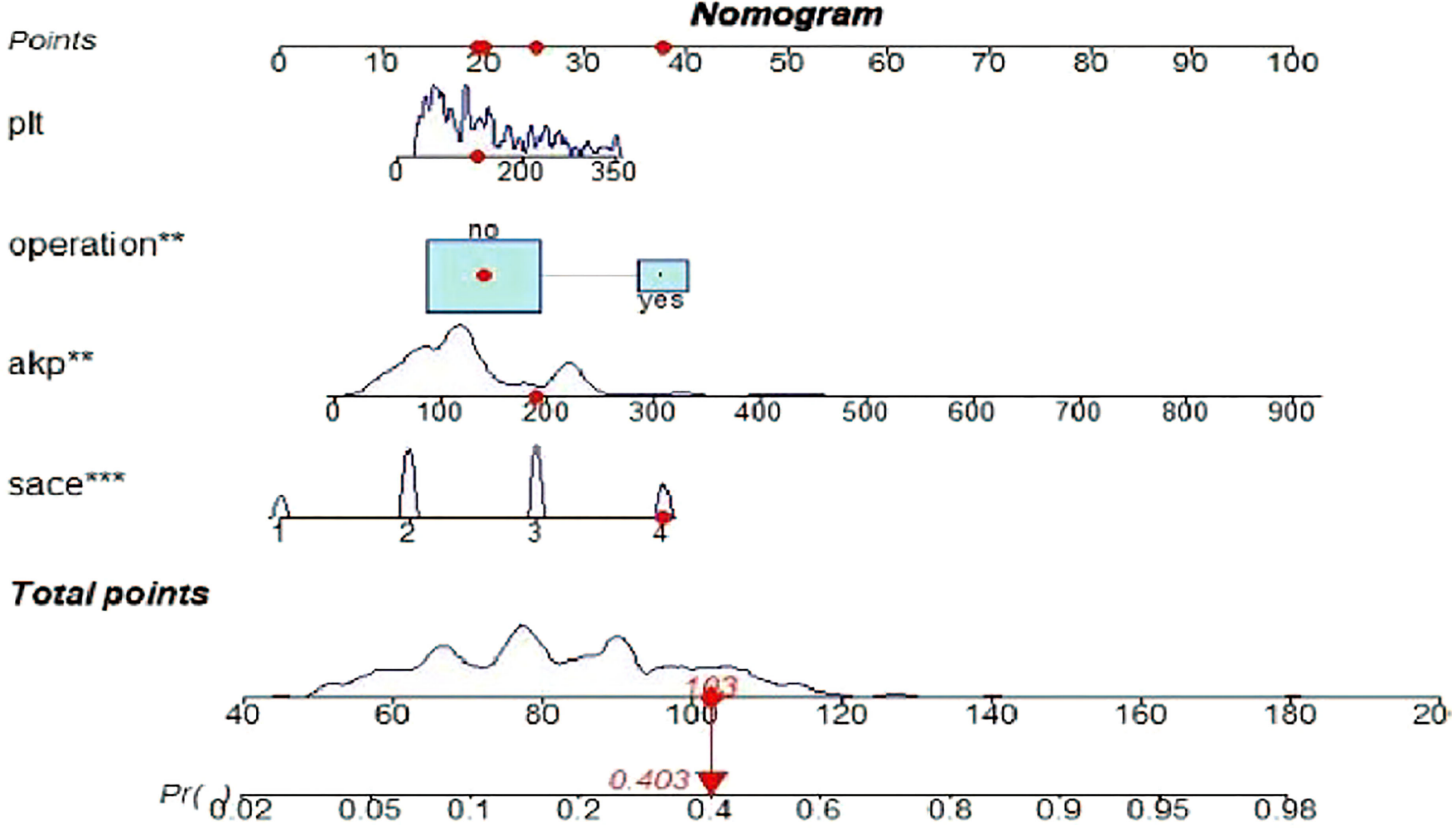
Risk nomogram model for the prediction of bile duct injuries after DEB-TACE in patients with HCC. **, *** refers to the contribution weight.

**Figure 5:**
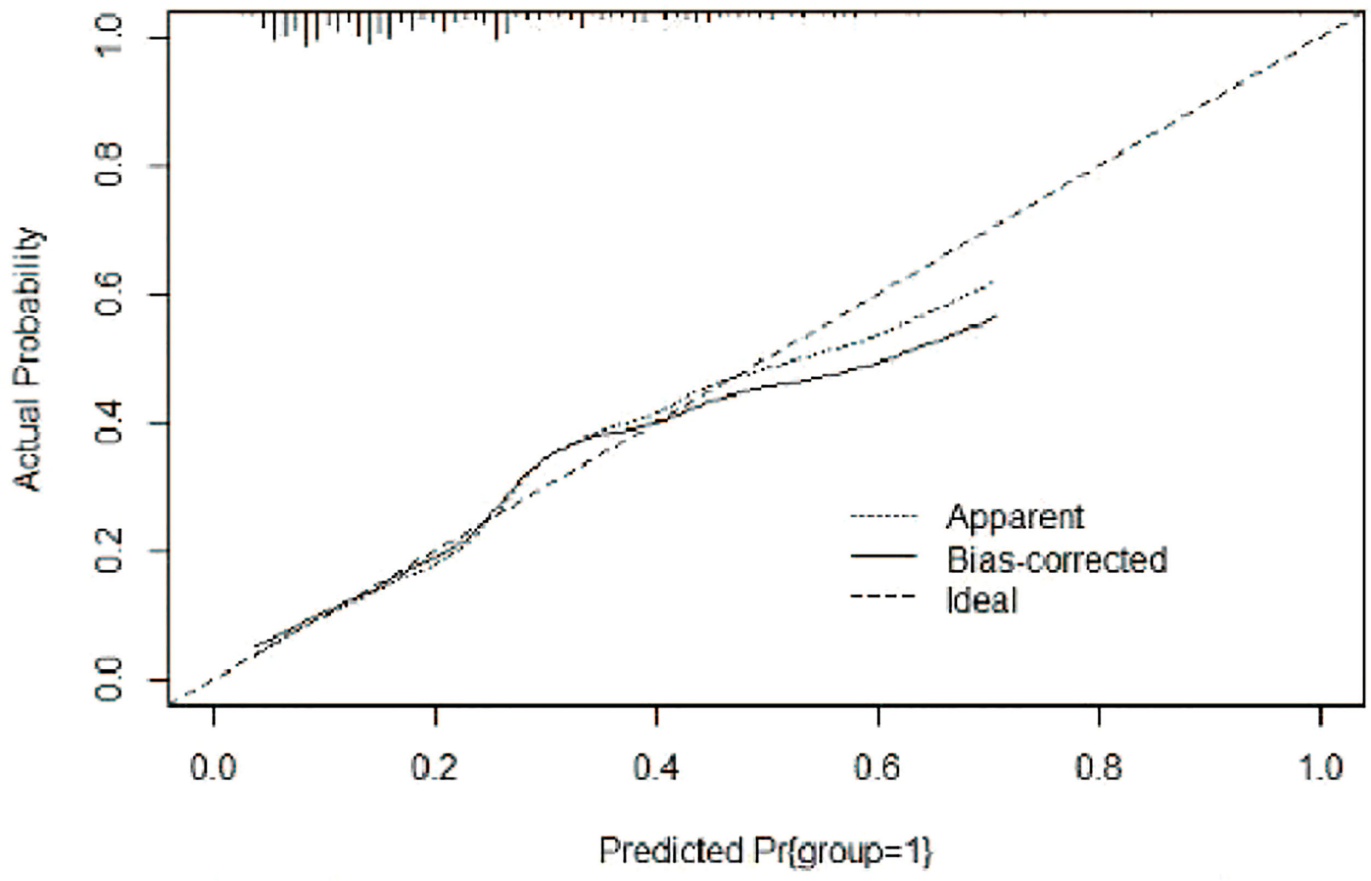
Calibration curve of the risk nomogram model for the prediction of bile duct injuries after DEB-TACE in patients with HCC.

**Figure 6:**
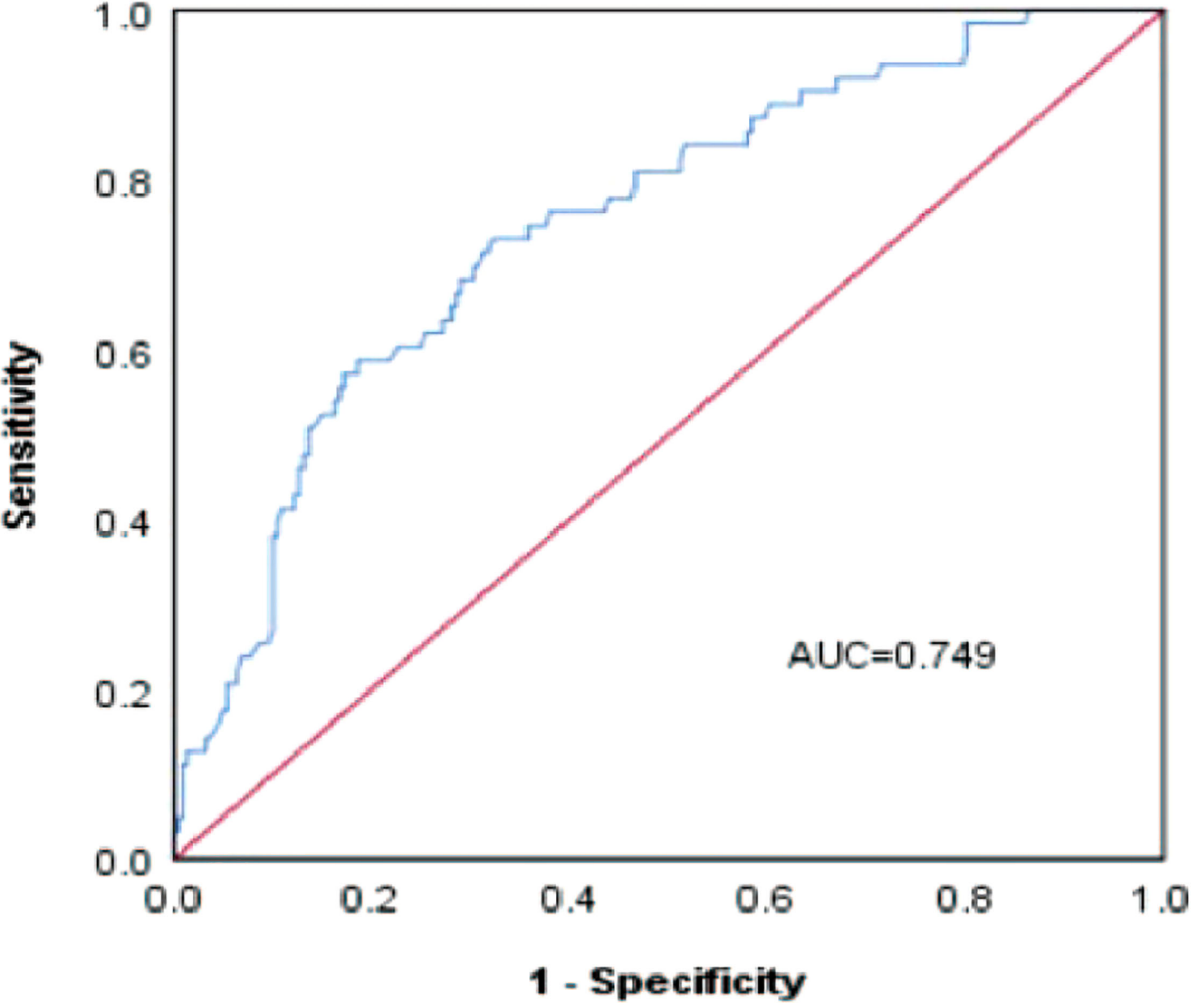
ROC curve of the risk prediction model for the prediction of bile duct injuries after DEB-TACE in patients with HCC.

**Figure 7:**
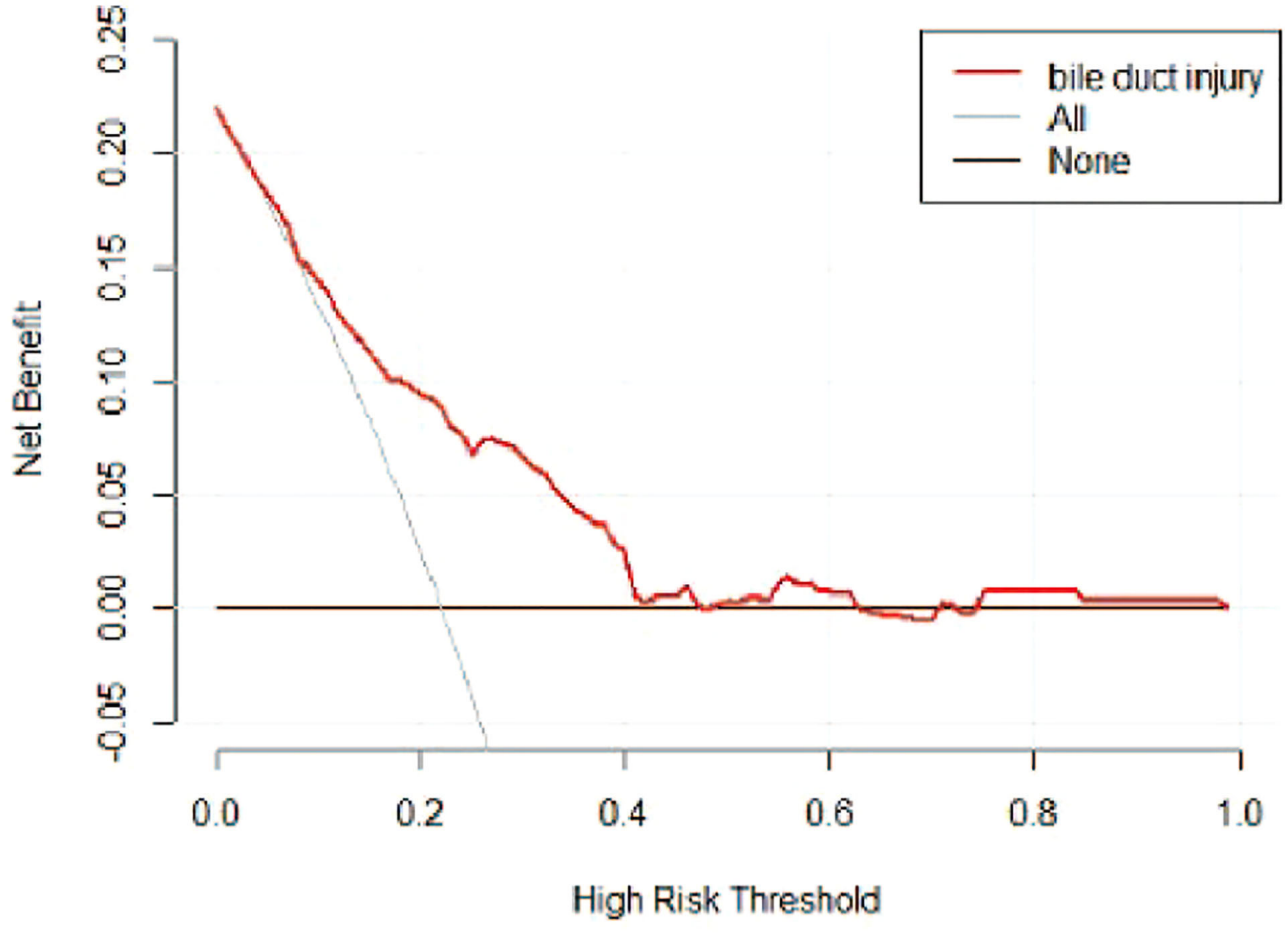
DCA of the risk prediction model for the prediction of bile duct injuries after DEB-TACE in patients with HCC. The horizontal line (i.e., the None line) represents that no patient had a bile duct injury, while the gray curve (i.e., the All line) represents all patients who had a bile duct injury.

**Figure 8:**
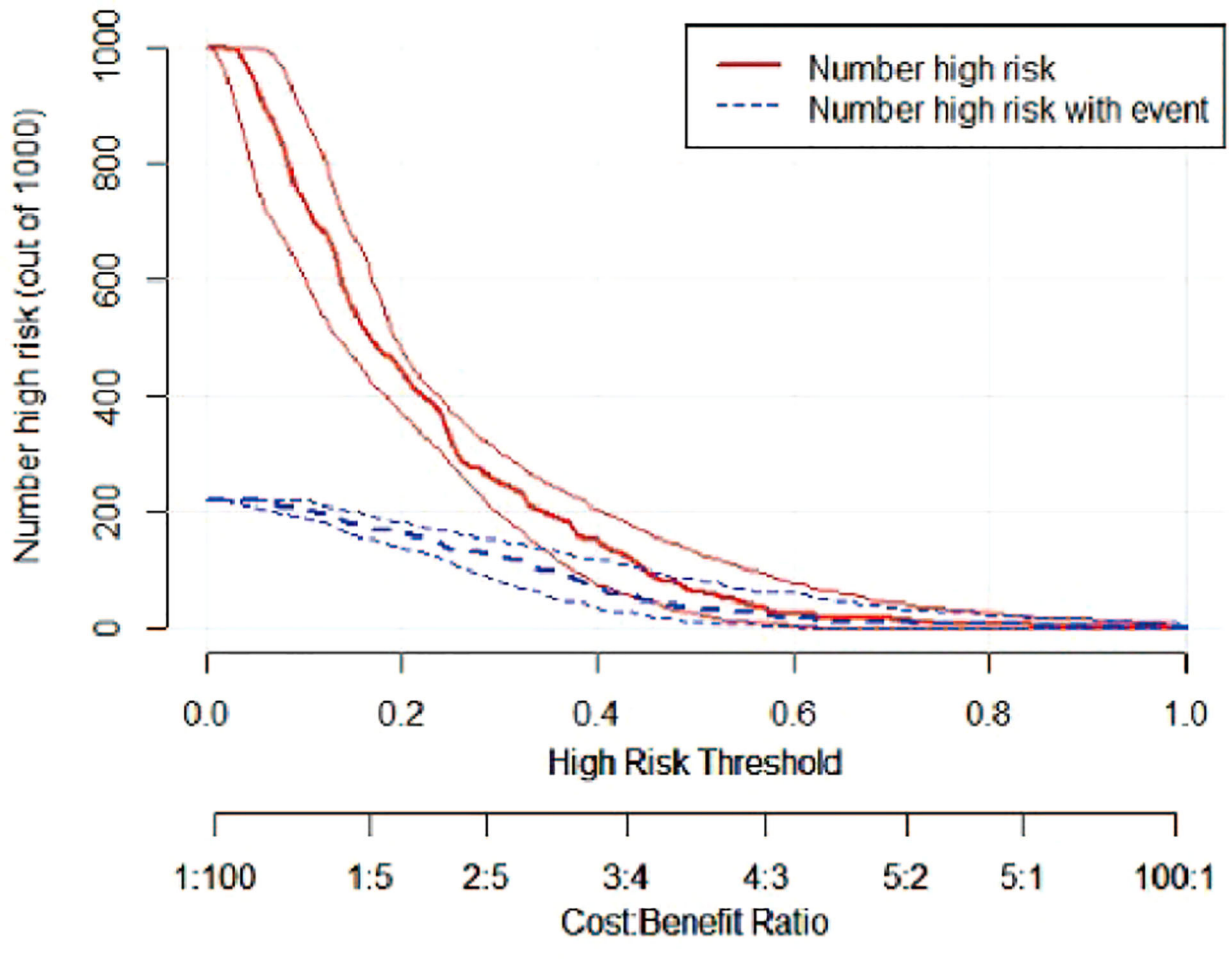
CIC of the risk prediction model for the prediction of bile duct injuries after DEB-TACE in patients with HCC. The red line (i.e., number of high-risk patients) represents the number of patients classified as positive by the model at each threshold probability, whereas the blue curve (i.e., number of high-risk patients with an outcome) represents the number of patients with true positives at each threshold probability.

A risk nomogram model was established based on the history of hepatectomy, SACE level, AKP, and PLT count (**Figure 4**). The nomogram was validated using the bootstrap method, showing consistency between the predicted and experimental values (**Figure 5**), and the model performed well in the Hosmer-Lemeshow goodness-of-fit test (χ^2 =^ 3.648; P=0.887), indicating its superior predictive ability. The ROC curve of the risk prediction model for predicting bile duct injuries after DEB-TACE among patients with HCC is shown in **Figure 6**, with an AUC of 0.749 (95% CI, 0.682–0.817), an overall accuracy of 69.01%, a positive predictive value of 73.02%, a negative predictive value of 67.87%, a sensitivity of 73.0%, and a specificity of 67.90%, suggesting that the nomogram model had good accuracy and discrimination. DCA in **Figure 7** shows that when the threshold of the model was set within the range of 0.1–0.6, the decision curve was located above the None and All lines, indicating that when the prediction model was used to make clinical decisions, a greater net benefit rate of the population could be obtained compared with the prediction scheme of “all bile duct injuries” or “none bile duct injuries,” further proving that the model had a high value of clinical practical application. In addition, CIC was drawn based on the DCA, which showed that the cost and benefit ratios were within the acceptable range (**Figure 8**), further suggesting that the model had high clinical practicability.

## Discussion

Since first reported in 1985 by a Japanese scholar, Masatoshi et al. (19), bile duct injury caused by TACE has been successively recorded worldwide. In 1993, Kobayashi confirmed through autopsies that the branches of the hepatic artery were embolized after TACE, resulting in bile duct necrosis (20). In 2001, Kim reported that ischemic bile duct injury after TACE for liver cancer is a serious complication (16). In 2008, Bang reported that repeated TACE could cause ischemic biliary stricture (6), and Guiu et al. in 2012 reported that a higher incidence of bile duct injuries was observed after DEB-TACE compared with c-TACE (9). With poor symptom specificity and difficulty in clinical diagnosis, once the ischemic bile duct injury is complicated by an irreversible bile duct injury, the clinical prognosis will be poor, and TACE has to be terminated in time. Therefore, the prevention and prediction of the occurrence of such complications have become the focus of recent research.

Generally, the primary etiology of bile duct injury after TACE is ischemia of the artery supplying the bile duct (16, 21). Several studies simulated bile duct ischemia by ligating the hepatic artery in rats and concluded that impaired bile duct function resulted in increased expression of the apoptotic bile duct and apoptotic cholangiocyte genes in liver tissue sections (22). Biliary artery ischemia can lead to necrosis and shedding of biliary epithelial cells, followed by bile duct dilatation and/or stenosis, bile retention and/or spillage, and finally, biloma or other irreversible injuries (23). The incidence of bile duct injury after DEB-TACE in our study was approximately 22.2% (63/284), which was lower than the 33% incidence rate reported in a previous study (10). This higher incidence may be explained by the presence of comorbid liver cirrhosis among our patients with HCC, as the proliferation, dilatation, and formation of a microarterial-portal shunt of the capillary plexus around the bile duct in cirrhosis acts as a compensatory mechanism for liver tissue ischemia, which could make it resistant to bile duct injury after TACE to a certain extent (6).

Among the 63 patients with a bile duct injury in this study, the incidence of intrahepatic and hilar bile duct injuries was 84% (53/63) and 16% (10/63), respectively, indicating that the former was more common and might be closely related to the biliary vascular anatomy. The bile duct is mainly divided into small, interlobular, and large bile ducts (including the left, right, and common hepatic ducts), of which the large bile duct is the predilection site for bile duct injury. The bile duct primarily originates from the hepatic artery, which is graded in turn, and finally forms a special bile duct system called the peribiliary vascular plexus, i.e., double capillaries located both below and around the bile duct epithelium. In addition to the blood supply from the hepatic artery branches and anastomotic branches, the hilar bile duct can also be supplied by the gastroduodenal artery through the anastomotic branches (7, 13, 24). In most DEB-TACE sessions, superselective catheterization is used for hepatic artery embolization, which avoids the feeding artery of the hilar bile duct and further reduces the incidence of hilar bile duct injury.

The association between the number of TACE treatment sessions and the occurrence of bile duct injury remains controversial. It has been reported that patients undergoing multiple TACE sessions were prone to bile duct injuries (6). This may be due to the incomplete repair and re-embolization of injured vessels due to repeated TACE sessions, followed by gradual reduction of blood vessels supplying the bile duct, and finally, ischemic injury. However, it has also been demonstrated that bile duct injury occurs in patients with HCC treated with one TACE session (9). In this study, we found that bile duct injuries occurred after an average of three DEB-TACE treatment sessions; therefore, we recommend that patients with HCC who underwent more than two or three DEB-TACE treatment sessions should be carefully monitored for the occurrence of bile duct injuries.

Currently, bile duct injury after DEB-TACE is caused by a combination of factors rather than a single factor. The results of the univariate analysis in this study suggested that age, history of hepatectomy, tumor burden group, SACE level, bead diameter, AKP, GGTP, and PLT count were associated with bile duct injury after DEB-TACE. The results of the logistic multivariate regression analysis showed that the independent risk factors for bile duct injury after DEB-TACE include a history of hepatectomy, SACE level, AKP, and PLT count.

Our results showed that a higher proportion of patients with a bile duct injury had a history of undergoing surgical resection for liver cancer than those without a bile duct injury, and some bile duct injuries were caused by DEB-TACE due to residual hepatectomy margins or microvascular invasion. In contrast, the surgical resection of HCC results in the modification of the intrahepatic bile duct supply system, which reduces its supply vessels while losing its siphon effect. TACE may cause the embolic microspheres to enter the normal liver parenchyma, increasing the probability of bile duct injury to a certain extent. However, during surgical resection, the hepatic artery and blood vessels of the bile duct are blocked, resulting in preliminary ischemia of the bile duct. If TACE is performed again to inject the embolic agent, it may lead to further bile duct ischemia and, finally, ischemic necrosis. Therefore, a history of hepatectomy may be a risk factor for bile duct injury.

Kobayashi et al. (20) performed autopsies on patients with HCC who underwent TACE treatment and revealed a reduced or even disappeared non-necrotic peribiliary capillary network near the biloma, as well as some peripheral vascular embolism and liver parenchymal atrophy. Malagari et al. (25) have reported that the degree of embolism was a significant risk factor for bile duct injury after DEB-TACE, which was consistent with our results, which demonstrated that the SACE level (OR, 1.832; 95% CI, 1.258–2.667; P <0.05) at the embolization endpoint was an independent risk factor for bile duct injury. Specifically, in the stratified analysis of SACE levels, it was found that when the embolization endpoint was SACE II, the proportion of bile duct injuries was significantly lower than that in patients without bile duct injury (9 injuries, 14.28% vs 90 cases, 40.72%; P <0.05). When the embolization endpoint was SACE III, there was no statistically significant difference between the two groups (P >0.05). However, in patients with SACE IV at the embolization endpoint, the proportion of bile duct injuries was significantly higher than that in patients without bile duct injuries (19 injuries, 30.16% vs 28 cases, 12.67%; P <0.05). The above results indicate that the more thorough the degree of embolization, the higher the probability of bile duct injury. With a higher SACE level, the embolic agent is more likely to enter the supply vessel of the embolized bile duct, resulting in biliary ischemic injury. To improve the tumor response rate and reduce bile duct injury, we recommend using SACE II or III rather than SACE IV as the embolization endpoint.

Laboratory tests for AKP, GGTP, and total bilirubin demonstrated good clinical significance in monitoring concurrent bile duct injuries. This study found that AKP and GGTP levels were significantly higher in patients with a bile duct injury than in those without a bile duct injury, and logistic multivariate regression analysis suggested that elevated AKP was an independent risk factor for bile duct injury (OR, 1.005, 95% CI, 1.001–1.010; P <0.05). Yu et al. (26) believed that significantly elevated AKP was more sensitive than CT imaging in predicting bile duct injuries. Therefore, we suggest that close attention should be paid to the possibility of bile duct injury in patients with higher AKP.

This study found that PLT counts in patients with a bile duct injury were significantly higher than those in patients without a bile duct injury through both univariate and multivariate regression analyses (P <0.05). Combined with the fact that most patients included in this study had cirrhotic portal hypertension and hypersplenism, the decrease in PLT count was closely related to the degree of hypersplenism. This conclusion again illustrates that cirrhosis is a protective factor against bile duct injury (9, 21). HCC patients with high PLT counts who underwent prompt DEB-TACE should be carefully monitored for the occurrence of bile duct injuries.

A nomogram has a high clinical practice value as it collates statistically significant risk factors according to the results obtained from the multivariate regression analysis. Based on the pre-calculated proportion, it uses lines with a scale to clearly describe the relationship between each risk factor in the model, such that the results of the logistic regression analysis are visualized and intuitive (27). A nomogram can be applied in clinical settings by adding the scores corresponding to various independent risk factors, and the total score corresponds to the risk prediction value referring to the incidence of specific events.

We established a risk nomogram based on the history of hepatectomy, SACE level, AKP, and PLT count. The nomogram was validated by the bootstrap method, showing consistency between the predicted and experimental values, and the model performed well in the Hosmer-Lemeshow goodness-of-fit test (χ^2 =^ 3.648; P=0.887). The ROC curve of the risk prediction model was plotted, with an AUC of 0.749 (95% CI, 0.682–0.817) and an overall accuracy of 69.01% (positive predictive value, 73.02%; negative predictive value, 67.87%; sensitivity, 73.0%; specificity, 67.90%), indicating favorable accuracy. In addition, DCA and CIC revealed a high clinical value and practicability of the model. Hence, our nomogram could perform individualized prediction of bile duct injuries after DEB-TACE in patients with HCC.

The prediction model of this study showed that patients with HCC treated with DEB-TACE had a higher probability of postoperative bile duct injury if they had a history of hepatectomy and higher PLT count, AKP, and SACE levels. For example, an HCC patient treated with DEB-TACE with no history of hepatectomy (20 points), PLT count of 180 × 10^9^/L (20 points), SACE IV (40 points), AKP of 200 U/L (23 points), will have a total score of 20 + 20+40+23 = 103 points, and the calculated predictive value will be 0.403, i.e., this patient has a 40% possibility of bile duct injury (**Figure 4**).

This study had some limitations. First, the retrospective study design might have incorporated information bias, and limited our selection of related variables. Second, only internal validation was used. Although there are many studies suggesting the value of internal validation (28, 29), external validation is still a more reliable way to validate models. therefore, an independent external validation cohort is needed in future studies. Third, considering that our treatment regimens were quite similar, we did not include risk factors such as chemotherapeutic drugs, which could also lead to bile duct injury after TACE. Future studies should include patients who underwent both transcatheter arterial embolization and TACE.

In summary, bile duct injury in patients with HCC treated with DEB-TACE was caused by multiple factors rather than a single factor. Based on the history of hepatectomy, SACE level, AKP, and PLT count, the nomogram prediction model established in this study had a good fitting degree and prediction efficacy with high clinical value and practicability.

## Data availability statement

The datasets presented in this study can be found in online repositories. The names of the repository/repositories and accession number(s) can be found in the article/**Supplementary Material**.

## Ethics statement

The studies involving human participants were reviewed and approved by the institutional review board of Shenzhen People’s Hospital. Written informed consent for participation was not required for this study in accordance with the national legislation and the institutional requirements.

## Author contributions

(I) Conception and design: JG, JK. (II) Administrative support: JK. (III) Provision of study materials or patients: JG. (IV) Collection and assembly of data: JG, XZ. (V) Data analysis and interpretation: JG, JK. (VI) Manuscript writing: All authors. (VII) Final approval of manuscript: All authors.
